# Flourishing Through Music Creation: A Qualitative Investigation of the *Lullaby Project* Among Refugee and Incarcerated Communities

**DOI:** 10.3389/fpsyg.2021.588905

**Published:** 2021-10-22

**Authors:** Sara Ascenso

**Affiliations:** ^1^School of Academic Studies, Royal Northern College of Music, Manchester, United Kingdom; ^2^Centre for Performance Science, Royal College of Music, London, United Kingdom; ^3^Learning Enhancement Unit, Trinity Laban Conservatoire of Music and Dance, London, United Kingdom

**Keywords:** music, flourishing, wellbeing, refugee, prison, community music

## Abstract

The *Lullaby Project* is an innovative model developed to support vulnerable groups through community-based music creation. It pairs expectant and new mothers with professional musicians, to create a lullaby for their children. This paper presents an investigation of the project’s pilot implementation in the United Kingdom, bringing together musicians from the Royal Philharmonic Orchestra, refugee mothers and inmate fathers from a central London prison. The research aimed to understand how the *Lullaby Project* was experienced, focusing on the potential areas of perceived change linked with the concept of mental health as flourishing. Participants (*N*=12) took part in semi-structured interviews and kept daily notes to aid recollection of the sessions in the interviews. Interpretative phenomenological analysis was adopted as the research approach. Participants considered the project to carry significance for them in three key areas: (1) wellbeing, through a strong sense of accomplishment, meaning and connectedness, and the experience of positive emotions; (2) proactivity, promoting initiative, both musical and relational; and (3) reflectiveness, stimulating perspective-taking and positive coping mechanisms. The *Lullaby Project* offers an effective model towards promotion of flourishing among vulnerable groups, and the results make a strong case for its implementation.

## Introduction

Community-based music projects have been reaching a progressively wider set of contexts and are leading to a promising body of research highlighting their potential to promote positive change. Findings have been unanimous in emphasizing the role of community music making in fostering key components of psychological flourishing, such as engagement (Cohen et al., 2006; Davidson, 2011), a sense of accomplishment (Perkins and Williamon, 2014), purpose, autonomy, control, social affirmation (Creech et al., 2013), and reducing anxiety (Hars et al., 2014) and loneliness (Koga and Timms, 2001; Cohen et al., 2006). This has been true both across general population samples and with vulnerable groups.

Recent research with mental health service users focused on group drumming found significant positive change on areas such as agency, a sense of accomplishment (both general and specific to musical goals), engagement, self-concept, and social wellbeing, as measured by qualitative inquiry (Perkins et al., 2016; Ascenso et al., 2018). The project also evidenced enhancement in quantitative indicators of general wellbeing, accompanied by reduction in depression and anxiety (Fancourt et al., 2016). Similar initiatives have also supported the psychosocial rehabilitation of psychiatric in-patients (Tague, 2012). Other reports have emphasized community-based music as an effective complementary tool for addiction treatments, by promoting the reduction of alienation and self-centeredness, through connectedness with self and others (Winkelman, 2003). These activities have also shown high potential to create a sense of community in under-privileged neighborhoods (Camilleri, 2002) and increase social learning outcomes for at-risk youth (Wood et al., 2013).

The *Lullaby Project* is another example of a promising intervention aimed at strengthening vulnerable communities through music-making. The initiative offers an innovative format of a 3-day workshop, inviting expectant and new mothers to work with professional musicians to create and record a personal lullaby for their children. It was launched at the Jacobi Medical Center (NYC), by Carnegie Hall’s Musical Connections team, and has been running for 10 years. It has now extended significantly across the United States, reaching mothers in hospitals, homeless shelters, schools, and correctional facilities. This paper presents the results of its pilot implementation in the United Kingdom, with two highly vulnerable groups: refugees and prisoners. Before introducing the project design, a closer look at the evidence base suggesting the potential of art-related interventions with both populations is explored.

### The Case of Migration

Arts-based community projects have proven highly valuable toward promoting integration and psychological flourishing of migrants, especially refugees. Migration predisposes individuals to a higher degree of physical and psychological vulnerability, both as a result of the pre-migration stressors as well as the challenges that acculturation, adaptation, and eventually transit can bring. Frequently, the migration process is also linked with trauma (Montgomery, 2011).

A review of 72 studies by the World Health Organization (WHO) (Bradby et al., 2015) points to a higher prevalence of mental distress among refugees compared with non-refugees, and greater risk for asylum seekers in relation to refugees. In the case of trauma, there is increased vulnerability for depression, anxiety, substance abuse, general distress, fear, deep sadness, guilt, anger, and impaired cognitive and affective functioning (Murray et al., 2008; Montgomery, 2011). This is often linked with intrusive thoughts about the traumatic events, avoidance, emotional numbing, and/or hyper-arousal. Also to consider are the potential risk factors for mental illness that resettlement can bring. Crucially, the loss of meaningful social roles and important life projects, lower levels of daily activity, and social isolation are evidenced to determine worse outcomes when integrating in the new community (Murray et al., 2008).

A report by the United Nations High Commissioner for Refugees Policy Development and Evaluation Service (Andemicael, 2011) has presented a review establishing strong evidence on the promise of artistic engagement as a means for achieving holistic human flourishing in refugee contexts. Previous accounts have highlighted the expressive potential of the arts toward helping migrants build new narratives, develop a sense of continuity, enhance meaning, and restore identities (Frater-Mathieson, 2004). This adds to the evidence of a positive impact of the arts toward inclusion and connectedness (Saether, 2008), meaningful community reintegration, resilience and the overcoming of stigma (Harris, 2007), communication, reduced stress, and a positive negotiation between a meaningful connection with the new country and the bond with the country of origin (Jones et al., 2004; Pesek, 2009; Lederach and Lederach, 2010). Musical engagement, in particular, has been found to promote inclusion and growth of newly arrived migrants, through fostering a sense of belonging (Marsh, 2012).

### The Case of Incarceration

Evidence on the potential of arts programs to tackle the psychological challenges inherent to incarceration is also growing. Despite a lack of recent data, survey studies have consistently described higher rates of mental illness in prison groups when compared to the general population, with an increase in recent years [see Hiday and Ray (2017) for a review]. Recent accounts have described estimates of around 49% for risk of anxiety and depression (National Audit Office, 2017), with other reports pointing to overall prevalence rates as high as 90% (e.g., Singleton et al., 1998; Lynch et al., 2014; Fazel et al., 2016; House of Commons Committee of Public Accounts, 2017). However, there is not a shared set of criteria across studies. Some reports adopt a rather broad definition of mental illness, and despite the unequivocally concerning figures, any comparisons need to be considered with extra care. Side-by-side with identifiable mental illness, qualitative evaluations have highlighted a wide array of concurrent, interwoven, issues that prison populations face, with a detrimental impact on psychological functioning, such as learning difficulties, poor social skills, debt, diminished sense of self-worth and loss of identity, aggression, bullying, fear, trauma, and poor physical health (Durcan, 2008). Additionally, being in prison can exacerbate pre-existing challenges, particularly due to a reduced sense of agency and meaningful activity, insecurity about the future, isolation, and social withdrawal. The prison setting has also been highlighted as typically leading to the development of hypervigilance, distrust and suspicion, alienation, and psychological distancing (Haney, 2012).

The role of artistic engagement in promoting flourishing among prison populations has also been evidenced. A review focused on arts projects in incarceration between 1997 and 2003 has highlighted the positive impact of the arts across central areas of functioning, such as self-confidence, social cohesion, and adaptive coping mechanisms, along with the crucial reduction in recidivism (Hughes et al., 2005). This added to previous reports evidencing an impact on general behavior and emotional control (Dawes, 1999; Brewster, 2014), stress reduction (Peaker and Vincent, 1990), social competence, initiative and artistic skills (Cox and Gelsthorpe, 2008; Brewster, 2014), hope, self-esteem, motivation, and positive relationships (Cartwight, 2013). An extensive qualitative investigation of a set of arts-based projects in UK prisons (Bilby et al., 2013), including a substantial number of music initiatives, suggested there are strong foundations to assume arts in criminal justice as an area of considerable significance and innovation. Key areas of change enabled by such activities include increased positive affect, connectedness engagement, achievement, and the development of positive identities. All these are considered to be at the core of the desirable developments toward behavioral change and desistance from crime that educational projects in prisons aim for.

### The *Lullaby Project*

The *Lullaby Project* exposes participants to a high-quality creative musical experience. Through a focus on the parent–child bond, it intends to facilitate a safe and fruitful space for emotional processing and expression to promote wellbeing. The project is built upon musician-participant dyads, and its format typically includes three sessions and one performance. On session one, there is a creative workshop-like encounter, where the musician and the participant write a first draft of the lullaby. The composition process is driven by the participant (both regarding the music and lyrics) and facilitated by the musician. Next, each song is arranged by the musical team. On a second session, the participants receive the arrangement and work on editing toward creating the final version. This is typically followed by a final sharing session where the lullabies are presented to the wider group of participants and their children. Finally, when possible, there is a performance to a general audience. High-quality recordings of their personal lullaby are given to each participant to keep.

The specific case of the role of music in optimizing the maternal relationship and wellbeing, at the core of the *Lullaby Project*, has also received wide attention in recent research. In particular, community-based projects focused on mother–child dyads have looked at the impact of singing on reduction of maternal stress, positive neonatal and infant behavior, language development, and the building of secure attachment. Children’s attachment style may be negatively influenced by any factor that may hinder the mother’s ability to attend to their needs, such as high levels of stress (Bowlby, 1969; Ainsworth, 1973; Miller, 2016). Music can play a very important role in this context, through strengthening emotional experiences, expression and communication, and contributing to the mother’s wellbeing. A recent systematic review has highlighted the effectiveness of music-based interventions in reducing stress levels and anxiety among expectant women (van Willenswaard et al., 2017). Maternal singing has been especially emphasized as a unique source of communication, and lullabies are of particular importance due to their repetitiveness, simplicity, slow tempo, and soothing effects. Singing lullabies has been evidenced to improve maternal–infant bonding, with positive effects on neonatal behavior and maternal stress (Persico et al., 2017). A qualitative evaluation of a lullaby-based project (Baker and Mackinlay, 2006), making use of traditional lullabies, highlighted how the process potentiated the mothers’ deeper understanding of their babies’ responses and enhanced feelings of motherhood. The *Lullaby Project* in the United States has also led to research evidence over the years, strengthening the case for lullaby-based workshops as a means for overall wellbeing and communication (Wolf, 2017).

This document presents the research findings from the qualitative investigation of the first pilot initiative of the *Lullaby Project* in a UK context. The project included mothers (as in its original design) and was also extended to fathers. It resulted from the appeal from both prisons and charities working with refugees toward receiving creative interventions to promote mental health, in combination with the intention of orchestras to expand their portfolio of community engagement projects. This led to a partnership between The Irene Taylor Trust – Music in Prisons, Praxis (a charity working with refugee and migrant women in East London), HMP Wandsworth (one of the country’s largest male prisons), and the Royal Philharmonic Orchestra. Given the challenges of migration and incarceration previously highlighted, and the potential of music-based initiatives to tackle some of the major needs among these communities, this study aimed to understand how participants experienced this project model. Of particular interest was to look at how the *Lullaby Project* was experienced in relation to participants’ mental health.

Despite the many reports linking music engagement with mental wellbeing, there is rarely a shared operational definition of what is meant by wellbeing across music psychology research, and reports tend to be typically presented in what appears to be a theoretical vacuum. The expressions “mental health,” “subjective wellbeing,” and “psychological wellbeing” are often used interchangeably. It is, therefore, useful to clarify what is meant by mental health in the context of this study.

### Mental Health as Flourishing

Most of Psychology’s history was dominated by a long-standing tradition of assuming that mental illness and mental wellbeing stood as opposites and that the treatment of conditions within psychopathology would result in a mentally healthy population. This, alongside an emphasis on the categorical classification of mental disorders, led to a tendency to focus on patients’ problems (Keyes, 2002; Corrigan, 2004). Following a gradual shift of paradigm, subscribed by the WHO and largely developed by Positive Psychology, there has been a progressive detachment from the medical model when looking at psychological health. “Being psychologically well” is now conceptualized as qualitatively different from the absence of mental illness. This paradigm deviates markedly from the focus on merely minimizing harm. As Seligman (2008) points out, wellbeing is defined by a combination of excellent status on biological, subjective, and functional measures. Investigating wellbeing, therefore, means assessing the presence of positive indicators of functioning, not merely the absence of negative ones.

Broadly, wellbeing entails feeling good and functioning well (Keyes and Annas, 2009). Wellbeing models have tended to emphasize either the former or the latter. Within a hedonic perspective, wellbeing has been equated as the optimal balance between positive and negative affect, along with perceived satisfaction with life. This has been coined as subjective wellbeing (Bradburn, 1969; Watson et al., 1988 Diener et al., 1999). From a eudaimonic approach, in turn, with a center on virtuous action and self-realization, wellbeing is best defined as the degree to which a person is fully functioning and self-actualizing (Waterman, 1993; Ryan et al., 2008). One of the most influential models of wellbeing was developed by Ryff (1989; Ryff and Singer, 2008), who put forth six dimensions: self-acceptance, environmental mastery, positive relations with others, personal growth, autonomy, and purpose in life. These elements are empirically distinct from hedonic components, and their investigation has strengthened the case for equating wellbeing as more than just “feeling good” or a subjective evaluation (Ryff, 1989; Keyes et al., 2002). In the context of this model, therefore, “psychological wellbeing” is defined by these six elements.

It has also been emphasized how wellbeing is not just a private phenomenon (Keyes, 1998). People belong to social structures and live out social roles. Social wellbeing, therefore, encompasses social functioning, standing as a distinct component from emotional and psychological wellbeing. Keyes (1998, 2002) proposes five dimensions of social wellbeing: social contribution, social integration, social actualization, social acceptance, and social coherence.

In recent years, models have converged toward operationalizing wellbeing as a combination of both hedonic and eudaimonic dimensions. The work of Keyes and Ryff led to a joint definition incorporating: (1) emotional wellbeing (positive affect and life satisfaction), (2) psychological wellbeing [consisting of Ryff’s (1989) six dimensions], and (3) social wellbeing [consisting of Keyes’s (1998) five dimensions]. Mental health is conceptualized as a syndrome of these positive symptoms of feeling good and functioning well, encapsulated in the term “flourishing” (Keyes, 2002). Flourishing consists of the presence of these symptoms. Languishing is conceptualized as their absence. In this study, mental health is conceptualized following Keyes’s (2002) proposal given that it is a theoretically driven model, it comprehensively incorporates hedonic and eudaimonic components, and it has received consistent empirical support, including cross-culturally and across the lifespan (Keyes et al., 2008; Westerhof and Keyes, 2010; Lamers et al., 2011; de Carvalho et al., 2016).

## Materials and Methods

The overarching question guiding this study was as follows: “how do participants make sense of the *Lullaby Project*?” The aim was to understand how individuals attributed meaning to the project, and ascertain potential areas of personal significance and positive change elicited by the sessions, as perceived by the participants. This will help to inform the reflection on the value of this project model to the specific communities under examination. Our question steers us to an in-depth inquiry, prioritizing subjective meanings and participants’ unique individual experiences, in context, pointing to the use of a qualitative methodology. The research focused on the experiences within the pilot implementation of the Lullaby Project’s model in the United Kingdom, hitherto unexplored in these contexts, calling for an inductive approach.

### Participants

The inclusion criterion for the study was to be a participant in the pilot implementation of the Lullaby Project in the UK for its entirety. The Lullaby Project included adults who were parents and were socially vulnerable, having endured adversity either through incarceration or migration. Initially, 17 participants took part, nine refugee mothers part of Praxis Community Projects and eight men serving a sentence at Wandsworth prison. All the mothers were recent migrants to the United Kingdom arriving from Bangladesh, Ethiopia, Eritrea, Ivory Coast, Nigeria, and Portugal. One mother was pregnant at the time of the study, and the remaining had young children. The men at the London prison were all British citizens and fathers. Ages for both groups together ranged from 22 to 49 years. During the project, three of the men dropped-out, due to prison transfer or release, as well as two of the women who were unable to attend the interview, leading to a final total of 12 participants providing a full dataset (seven women and five men). To preserve anonymity, fictitious names will be used.

### Procedure

Data were collected at all phases of the *Lullaby Project*. In-depth, face-to-face one-to-one interviews were conducted with all participants after the final performance. During the project, all participants were asked to maintain diary self-report notes after each session. This element functioned as a prompt for sharing the experience in the final interview, providing a complement to the retrospective recollection with a moment-by-moment recollection, to reduce memory bias. For this purpose, a one-page note form was delivered after each session, with three prompts for open comments: “Today’s session was…; Before the session I felt…; After the session I felt…” All participants were given the opportunity to write their diary notes in their native languages. All chose to reply in English. To complement the interview data and the participants’ reflection notes, the researcher was a non-participant observer in a sample of the project’s sessions as well as in the final performance, which allowed for observational data in the form of reflective notes for three full days of the project. Observation took place in the context of the sharing of experiences that accompanied the construction of the personal lullabies and the performance (where each song was introduced and explained).

A semi-structured plan was chosen for all one-to-one interviews. There was a small set of broad, open-ended questions (see Supplementary Material) that were used with flexibility, allowing for the content introduced by the interviewee to drive the interview. The interview aimed to grasp how the participants made sense of their experience of the project, and their perceptions on its personal significance. As the interviews were flexible and highly driven by the content brought by the interviewees, duration was also variable. Interviews lasted, on average, 45 minutes.

All interviews were recorded with permission and transcribed verbatim. In the mothers’ group, as was the case with diary notes, the participants were offered the possibility of being interviewed in their native language. Three mothers chose to do this partially. The researcher is fluent in two of the languages chosen, Portuguese and French, and it was possible to conduct these interviews as expected. The third mother chose to be interviewed in English and during the interview asked to use Amharic for part of her answer to two of the questions, translating herself as she spoke. The transcript was fully translated into English by an external translator before analysis.

This study was granted ethical approval by the National Offender Management Service (NOMS). All participants provided informed consent. No compensation was given in exchange for participation.

### Analysis

The goal was to investigate how participants made sense of the Lullaby Project, in context. Interpretative phenomenological analysis (IPA) was adopted, given its commitment to idiography, prioritizing the understanding on how individuals construct meaning and make sense of a phenomenon (Eatough and Smith, 2008; Smith et al., 2009). Besides having been used effectively across a wide variety of areas in psychological research (Reid et al., 2005; Brocki and Wearden, 2006), IPA has been found highly appropriate in recent studies exploring the experience of music community-based projects (Perkins et al., 2016; Ascenso et al., 2018).

The analysis proceeded in six steps and was conducted using NVivo 11 software. Each individual case was analyzed separately. First, each individual transcript, including interviews and diary entries, was closely read multiple times. This provided familiarity and a holistic perspective of the data. Second, points of interest and emergent meaning units were noted on the left-side margins. These were also labeled in NVivo. Third, the meaning units were clustered together based on similarity to form emergent subordinate themes. Data from the observation notes were used as an aid for interpretation. Fourth, for each individual, the subordinate themes were integrated into tables and a description of each one and their interconnections was noted. At this point, a table for each participant was built, representing the areas of change elicited by the project. Fifth, after each transcript had been closely scrutinized, individual tables were integrated into one overall table, capturing the study’s final superordinate themes and subordinate themes (see Table 1). Finally, as the themes for both the group of mothers and fathers were highly convergent, the two sets were merged into one overall table.

**Table 1:** Superordinate and subordinate themes encapsulating the areas of change elicited by *The Lullaby Project.*

Superordinate Theme	Subordinate theme
Wellbeing	1.1 Accomplishment
	1.2 Meaning
	1.3 Positive Emotions
	1.4 Connectedness
Proactivity	2.1 Musical initiative
	2.2 Relational initiative
Reflectiveness	3.1 Perspective
	3.2 “Beyond the tunnel-vision”

While remaining an inductive process fully driven by the content brought by participants, interpretation on what constituted accounts linked with flourishing was informed by Keyes’s proposal (Keyes, 2002). Emergent themes were regularly revisited within and across transcripts to ensure a valid representation of the raw data and acknowledging the importance of reflexivity in qualitative research.

## Results

IPA revealed three superordinate themes and eight subordinate themes, representing how participants made sense of the *Lullaby Project* (Table 1). In what follows, each superordinate theme is explored in relation to its constituent subordinate themes and example quotes that encapsulate their meaning are presented.

### Theme 1: Wellbeing

All participants reported the experience of the *Lullaby Project* in relation to perceived greater wellbeing. This was the strongest superordinate theme. Four dimensions were highlighted across accounts: (1) accomplishment, (2) meaning, (3) positive emotions, and (4) connectedness.

The first subordinate theme emphasized across all accounts was the experience of a high sense of accomplishment (subordinate theme 1.1), culminating in the final performance:

*Amazing, it’s very touching! When I got back to the cell I was feeling I have done something right for the right reason… […] I could just always remember the smile of my little man…* Richard.

Linked with this was the recurrent report of increased confidence, mentioned in relation to the process of getting out of one’s comfort zone and overcoming self-doubt at the start of the project.

*This project gave me confidence. From the very beginning, I had no idea about music and did not know how what we had discussed would change to music. […] When it was recorded and I listened to it at home, I felt that I had given something from the inside to my children and I was very happy.* Fatimah.

The feedback from the audience after the performance and the reaction of the participants’ children to the lullaby were highlighted as key ingredients fostering this sense of accomplishment. In the women’s group, there was additional strong positive feedback from peers within the project as well as from loved ones, including family members in the participants’ countries of origin.

*I told my friends and I called back home in Bangladesh and I said “I wrote for my children!” They say, “oh, in Bengali?” I said “No! English!!!,” I said “Yes, it’s English.” They say, “oh my God, you wrote an English song for your children? You are amazing!” […] “One day I’ll send you the CD!!!”* Preetha.

The contribution toward productivity, in a context which presents extra challenges around autonomy and personal growth, was also highlighted:

*Unfortunately, the thing with jail is you are very restricted so even if you have the best intentions to read a lot of books or do whatever, you are restricted to your cell where there’s not a lot. So that’s very unfortunate because a lot of people, like myself, want to do stuff. We regret that we are here in the first place and we want to come out with an improvement… You do not want to spend, for my case, it’s 15 and a half months I’ll be here, minimum, having made no progress or not accomplishing anything. So finding those opportunities to make progress are scarce in this jail […] I regret my mistakes. I accept my punishment, I’m not bitter or anything… I want to plan ahead and make sure this is a life lesson which it already was for me, definitely. But to be able to come up with something productive in jail is difficult […] I completely endorse the project because of those reasons. John.*

Closely linked with the experience of achievement, was a high sense of meaning as a result of the project (subordinate theme 1.2). The goal of the project was seen by all participants as a trigger for enhanced significance, representing the establishment of positive memories not only through revisiting important moments from their past, but also through creating new positive memories during the project. In the case of the women’s group, the theme of meaning was linked with the lullaby standing as a landmark for their resilience and a reminder of their process of struggle and at the same time of the hope they maintain, denoting a sense of purpose and direction in life:

*They will listen to it in the future, when our problems will be solved. Ah, then everything will be okay. Listening back to this music, we’ll be like: “wow… so, this is where I came from,” you know? This is the step I went through… And our children, as well, when they listen to that, they will know how much suffering mummy went through.* Ella.

For the men, the theme of meaning was linked with the lullaby standing as a proof of care for their children, despite their physical absence. For the participants who are not allowed to see their children, the sense of meaningfulness found in the project was built primarily upon the song standing as a powerful vehicle for communication. For one participant, who is allowed regular contact with his son, the gift of a lullaby was seen as a more significant way of connecting, beyond what is possible in the visits.

*I am trying to do it more for my son to have memories. I see him every week, or every second week he comes up, but he does not get anything from me… just to sit there, he will not eat something… […] and this is giving him more to remember […] this is little memories that he can remember. Because he feels that daddy’s left him and daddy does not come home. So what can you do?* Richard.

Linked with an increased sense of achievement (subordinate theme 1.1), one participant also highlighted enhanced meaning for being able to give to others and play his part in the overall group achievement. After having experienced frequent educational programs in prison targeted at promotion of specific skills, which, in his words, “were trying to fix what needed to be fixed,” this was the first time he perceived receiving attention for something of positive value he could contribute with – his personal lullaby. Echoing this sense of contribution and artistic identity, one of the mothers mentioned having forgotten she was a refugee while in the sessions.

Finally, besides providing a general sense of significance, the project brought purpose to everyday routines. One inmate, for example, shared how the process of song-writing positively punctuated his schedule during the prison’s lockdown periods, bringing a meaningful structure to his days.

Besides promoting a sense of accomplishment and fostering meaningfulness, the *Lullaby Project* contributed to the participants’ wellbeing through also eliciting positive emotions (subordinate theme 1.3). The sessions were reported as a source of joy, satisfaction, surprise, enthusiasm, gratitude, and awe. The moment when the participants’ heard their lullaby being played for the first time and the final performance were pointed by all as the emotional highlights. The process of writing the song was emphasized as a fruitful space for both expressing and processing a wide range of emotions. This included negative emotions, such as sadness and regret, in a process which enabled a sense of relief, peace, and freedom. The experience of positive emotions was mentioned both in relation to the actual days of the sessions and performance, and also linked with a sense of anticipation during the time leading up to them.

*It’s different if others sing… we are the ones singing for our baby here, this is something very different… More moving… you feel happy inside, you know? Yeah. I feel happy!* Sophia.

*I’m always excited before I come to the group at Praxis, but now I’m more excited because of the music! It helps me! Every week I wake up quick and get ready, my children come… I’m so excited to come. It makes me happy!* Preetha.

The project’s bonding effect, stimulating a sense of connectedness (subordinate theme 1.4), was another strong theme emerging from the data, closely linked with the perception of the sessions as a fruitful space for emotional expression. Both groups of participants highlighted how, through encouraging emotional communication, the project strengthened their relationship with their children, contributing to the development of a stronger bond.

*It helps in our connection a lot. Because first I was using only his name when I sang to him, but now with this lullaby, it’s some words that come from my heart with the sound that I always make with his name…Yeah. My other son, David wasn’t there, but I’m sure when he will listen, he will feel… he will recognise that…* Sophia.

In the women’s group, besides promoting a greater sense of connectedness through increased expression with the children, the lullabies also functioned as a means for increased empathy, deepening the sense of belonging among the mothers themselves, especially in the case of a sub-group who share the same house. They recorded each other’s songs and sung them regularly to one another and to each other’s children. The process of writing the songs also marked their interactions and led to meaningful sharing of personal narratives.

*And we know which song we should sing to our children, […] Preetha’s song…. I’m very close to her. So, her song touched me. Seriously, I think it’s the lady that helped her to blend those words. She did a fantastic job because, as a person who knows Preetha, when she starts singing, I feel… every word, I feel them inside me. I cried that day. When I see [Preetha’s children], I always sing that song for them. And she sings my song to my children…* Ella.

The women’s group also highlighted how the emotional expression leading to higher connection was fostered despite the barrier of language. Music was a vehicle for effective and strong expression within a group of highly varied linguistic backgrounds, encouraging social integration and acceptance.

In the men’s group, the theme of connectedness was particularly strong, as the contact of most participants with their children is highly restricted. For this group, the songs were recurrently mentioned as a means for communicating an important message (linked with the subordinate theme of meaning), and through that, functioning as a relational bridge in a situation where it is hard to develop the paternal bond.

*I thought it was a good way to connect with my kids, because it’s been an up and down relationship between me and them, and their mum. Previously I used to be using a lot of drugs so… it sort of spiralled a bit, and this is something for me to try and gain a bit of… what’s the word… relationship back…* Richard.

*I have not been in touch for a while… it’s hard to stay in touch when being in prison. It’s a powerful message to get across […] I have not seen them in such a long time… hopefully if I can get this sent to their address or in some way hand it over to their mum, it would be a great thing for them to be able to know that I am still there […] I’m constantly thinking about them and that they do still mean the world to me.* Adrian.

For the men, the theme of connectedness was also linked with the positive relationships established between them and the Royal Philharmonic Orchestra musicians. Most men mentioned how they valued the positive interactions with the team and how that contributed to a sense of acceptance and connection. In this context, one participant spoke of the project as representing the fruitful and unexpected encounter of two highly separate worlds: the world of professional classical music and the world of incarceration.

*I think that the Lullaby Project with the professional musicians is really cool because it then mixes two worlds […] they are quite polarised and not even just from a prison point of view but from a music point of view. You get a very certain type of person that is heavily involved in classical music as opposed to pop, pop modern culture music. That was really good, that was a good relationship as well!* James.

Overall, the project was experienced by all participants as a strong promoter of wellbeing through enhanced accomplishment, meaning, positive emotions, and connectedness. Wellbeing stood as the most prominent feature reported across accounts.

### Theme 2: Proactivity

The second theme accounting for how participants made sense of their experience of the *Lullaby Project* concerns proactivity, here used to mean self-initiated positive action. This theme appeared closely intertwined with the high sense of achievement (subordinate theme 1.1), meaning (subordinate theme 1.2) and the experience of positive emotions (subordinate theme 1.3). Participants experienced the project as a trigger for new plans, general engagement, and proactive behaviors, manifested both within the sessions and beyond. This was always mentioned linked with a perception of some degree of personal improvement and growth. The high commitment to the activities of the project itself was seen as a first sign of greater agency. The successful engagement throughout the project was highlighted as a starting point for a belief of competence that led to experimenting with other activities.

*It makes you believe you can do a bit more… now that you have done this, you can probably do some more things…* Ryan.

For some, the enhanced autonomous initiative was primarily music related (musical initiative; subordinate theme 2.1). This was highly emphasized by the women’s group, with participants sharing how they established new musical rituals with their children as a result of the sessions, as well as starting to intentionally share music with peers.

*Now [after the project], I sing for my children*. Preetha.

*I always sing to her…. I’m always singing, I’m always singing… I sing the chorus in Portuguese… “mummy’s baby, mummy’s baby” … I always sing it… Now with the CD, I’ll have our own music to play for Maria… I can give her a massage and listen to our song… every day.* Isabel.

Two of the men (one, soon to be released) shared their wish to start learning an instrument as a result of the project. Another participant mentioned his plan toward starting to listen to classical music, and three men suggested the lullabies would be showcased on the prison radio, a plan that was fulfilled soon after the research interviews were completed.

*I’m thinking of expanding my horizons… More so with the type of music, because I was born in the 70s so I’ve got this hip-hop/rap… so it’s natural you know, but this type of music is beautiful, I’m expanding… yeah expanding…* James.

The experience of enhanced proactivity through the project was also relationally oriented (relational initiative; subordinate theme 2.2). Participants highlighted further connection initiatives after the sessions, such as writing to loved ones to whom they had not written for some time or journaling about their relationship with their child.

*I wrote to my ex-girlfriend!! I wrote to her! And I thought about what was happening… and I wrote to her! And talked to her about the concert. And I sent my letter to her… And told her I that cannot explain it, and I said I did not think I would be able to do something like this… And I’m surprised with myself.* Ryan.

In the mothers group, participants also mentioned the excitement about the process of writing the lullaby as the starting point for re-connecting with their family members in their country of origin. There were also frequent accounts of intentions to develop new projects in the future, with a relational focus.

*It gave me an idea for the future. Because me too in the future, I want to be helping people. I want to do a lot of things. So, it’s very inspiring… it’s an idea, as well. Something new that I see, and I’ll keep it forever.* Ella.

One participant expressed how she has started to help charities, even if only with very small amounts of money, prompted by gratitude and the recognition of the impact of community work such as this. Another mother also expressed her wish to pay-it-forward, sharing her dream of being able to help others in the future through leading a replication of the project.

### Theme 3: Reflectiveness

Finally, the last emerging overarching theme concerns the reflection process triggered by the *Lullaby Project*. This theme appeared strongly intertwined with the subordinate theme of meaning (subordinate theme 1.2). Reflectiveness was consistently mentioned as one of the elements the project fostered, especially in relation to the development of a richer perspective about one’s life and one’s children (subordinate theme 3.1).

*It was a very interesting, reflective process […] the lullaby for your children naturally starts the reflection process.* James.

The process of translating their story and the narrative of their relationship with their child into the lullabies’ lyrics was mentioned as a source of insight into their own sense of resilience, triggering gratitude and strengthening hope and optimism about future goals.

*I could be sleeping around but at least I’m fine, my baby is fine… she’s very well, she’s very happy! I have achieved so many things. I have an opportunity! […] So many things I have received for my baby, I know it’s really hard but for me it’s really easy because of my baby. […] Later there will be joy!* Ade.

The theme of reflectiveness was also strongly mentioned with a focus on the process of coping or, in the words of one participant, “overcoming the tunnel vision” (subordinate theme 3.2). The “tunnel vision” was described as the tendency of trying to block thoughts about oneself or one’s story which can bring suffering and regret. The reflection triggered by the project was perceived as a vehicle toward reconciling this tendency with the longing for a new identity and greater connection. It allowed to process difficult thoughts in a safe, constructive way.

*Sometimes when you are in these sort of places, you try and block things out, like a tunnel vision of yourself. But yeah this project has made me think about my kids a lot, and at the end it brought me a lot of strength. […] Here in prison you try and block out the stress side of things […] Just get on with your day-to-day life. That’s how I deal with it… […] Try and not think too much about your kids as well… […] This project is a positive way of sort of solving that… of dealing with that.* Alexei.

In summary, the reported experience of all participants points to the *Lullaby Project* as a positive tool toward promoting flourishing. Closely intertwined with key wellbeing dimensions - such as a strong sense of accomplishment, meaning, positive emotions, and connectedness - was the increase in proactivity and reflectiveness. The project stimulated agency and initiative, both musical and relational, and allowed for a space of reflection, encouraging a richer perspective on life and the unblocking of avoidance mechanisms.

## Discussion

This research stands as a starting point towards affirming the *Lullaby Project*’s model in the UK context, in particular with refugee and migrant mothers and incarcerated fathers. One of the most exciting features of the results was the high convergence of themes across the two sub-groups. Adding to the existing evidence base from the *Lullaby Project* in the United States with mothers as participants, this allows to equally consider positioning fathers as potential partakers of future projects.

The *Lullaby Project* was experienced by all participants as a means to enhanced wellbeing. It triggered a strong sense of accomplishment, meaning, and connectedness, along with positive emotions. It was also experienced as a stimulus to proactivity, both musical and relational, across contexts. Finally, the *Lullaby Project* instilled reflectiveness, contributing towards a richer perspective in life and positive coping mechanisms. The three superordinate themes appeared highly interwoven and overlap with several of the crucial building blocks of flourishing proposed by Keyes (2002). Crucially, they also meet specific needs of the contexts of vulnerability under examination.

The fostering of a strong sense of accomplishment (subordinate theme 1.1) was voiced by all participants. Interestingly, this was not related to musical abilities or previous experience. Despite a wide diversity in levels of musical skill and varied degrees of previous exposure to song-writing, everyone was comfortable with the level of challenge and experienced a sense of competence, success, and satisfaction with both the creative process and the output. This echoes previous research highlighting the inclusivity of music-based community projects as one of the features potentially mediating its positive impact (Perkins et al., 2016). The project provided a safe space for individual choices, which allowed for artistic freedom and expression, while also ensuring the necessary resources and mentoring support. This sense of agency and environmental mastery, also evident in previous community-based project accounts (Ascenso et al., 2018), is of key importance for personal growth (Ryff, 1989; Keyes, 2002).

This experience of achievement also emerged as highly linked with proactive behavior (Theme 2). The strong ownership around the artistic output instilled by the project model could help explain the strengthened confidence which, in turn, seems to have potentiated self-directed, autonomous behavior revolving around musical and/or relational initiatives (subordinate themes 2.1 and 2.2). Here too, there seems to be a sense of personal growth, with the evidence of expansion and openness to new experiences in both groups.

The consistent reporting around increased agency is particularly encouraging. Personal stagnation, lack of productivity, and reduced levels of daily activity are commonly mentioned as risk factors for mental illness in prison contexts (Haney, 2012) and migrant communities (Murray et al., 2008) alike. The UK prison context in particular, despite benefiting from a wide variety of educational initiatives, has been highlighted for its passivity and lack of fruitful engagement (Durcan, 2008). The evidence of proactivity triggered by the project incorporated both individual and collective initiatives (e.g., the showcasing of the lullabies in the prison radio) and represented a starting point for a sense of strategic growth, which, if maintained, has the potential to translate into further adherence to educational projects and work-related activities.

Another strong theme emerging from the data was increased meaning. Interestingly, there was evidence of a positive impact across what have been proposed as the three dimensions of meaning (Martela and Steger, 2016): (1) purpose, through new goals and a sense of directedness; (2) coherence, with enhanced comprehensibility, reconciling past and present narratives as well as enhancing prospection about the future; and (3) significance, through a sense of the inherent value of one’s life story.

One of the distinctive elements of the *Lullaby Project*’s design is the centrality placed on individuality and autonomy in the process of creating. Both music and lyrics are driven by the participants, and meaningful self-expression of personal narratives is at the core of the composition process. In the mothers’ group, the songs themselves were musically diverse and often showcased their cultures of origin, either rhythmically, melodically, or both. The high respect for one’s individuality and narrative in the artistic process is key toward fostering the desired positive negotiation between new and old contexts of being. Promoting ways to sustain both the connection with the new country and the bond with the country of origin has been stressed as particularly important for successful adaptation in a process of resettlement (Murray et al., 2008) and seems to fit particularly well with this model.

Both migration and incarceration are experiences of high risk toward a sense of hopelessness and a consequent passiveness (Murray et al., 2008; Haney, 2012). Even when participating in effective educational programs, participants are often placed in a position of receivers of help, and addressed as so-called “target-populations.” In contrast, the engagement in such a person-centered artistic initiative, where the participants function as highly active creative agents, reinforces the crucial sense of personal contribution. The *Lullaby Project* dissolves hierarchy and instills artistic partnership, as was highlighted by one participant who mentioned it was the first time he received attention for what he can give, rather than for needing support.

Our results echo previous accounts of artistic engagement as a means to foster the building of new narratives about oneself and one’s history, helping also to develop a sense of continuity among groups who have experienced adversity (Frater-Mathieson, 2004). The personal lullaby often appeared as a bridge between past and present (e.g., through triggering the establishing of lost links with family members; through integrating elements of one’s culture of origin, such as indigenous musical features, along with lyrics focused on a hopeful future in the United Kingdom, etc.).

Adding to a sense of accomplishment and enhanced meaning, participants were also unanimous about the role of the *Lullaby Project* in eliciting positive emotions and connectedness. These two elements were often mentioned together. Both dimensions were expected, given their pervasiveness across community-based music project reports, particularly when high collaboration is involved (Perkins et al., 2016; Ascenso et al., 2018). Crucially, the accounts tapped into key elements suggested in Keyes’s (2002) model of flourishing: positive affect, positive relationships, social integration, and social acceptance. Through strong co-construction, the experience seemed to strip away variables with the potential to distance individuals from one another, such as cultural background, personal history, language, status, and position. It allowed for the display of some of the best shared human qualities such as empathy, compassion and understanding, triggering connectedness, through what one of the musicians referred to as “very raw emotion” after one of the sessions. The sense of belonging that the relational dynamics of the project sustained has also been highlighted as a key ingredient toward meaningful integration of migrants (Marsh, 2012) and offers promise to tackle the social withdrawal, alienation, and distrust that characterize prison contexts (Haney, 2012). Additionally, not only was there evidence of strong relational bonds within the project group, there were also accounts of increased relational initiative extended to other contexts (subordinate theme 2.2.).

Finally, besides the perceived changes in wellbeing, alongside increased proactivity, participants reported reflectiveness (Theme 3) as a main theme characterizing their experience of the *Lullaby Project*. The strongest thread within this theme was perspective-taking, denoting greater insight about one’s story, positive acceptance of the past and a link with meaning (subordinate theme 1.1.), through a sense of coherence. The project also allowed participants to address difficult thoughts in a safe, constructive way, in line with previous accounts (Hughes et al., 2005).

It can be argued that both the proactive behaviors (Theme 2) and the enhanced reflection (Theme 3) can stand simultaneous as perceived outcomes and potential mediators of the positive effect of music creation on individual flourishing. Tay et al. (2018) have recently emphasized the need to systematically address the potential mechanisms through which involvement in the arts and humanities may lead to flourishing. Mechanisms in this context are defined as “reactions to, or psychological experiences arising from, the modes of engagement and activities of involvement within the arts and humanities” (Tay et al., 2018, p. 218). The authors suggest four mechanisms as a possible framework to further understand this dynamics: immersion, embeddedness, socialization, and reflectiveness.

Immersion refers to the “immediacy that often attends engagement with the arts and humanities (…) One’s attention is captured, resulting in the experiencing of various levels of sensory and emotional states and first-order cognitions, often leading to a feeling of being ‘carried away’” (Tay et al., 2018, p.217). The report of one mother on how she forgot she was a refugee during the sessions is an example of how this sense of positive absorption was explicitly mentioned. The mechanism of embeddedness refers to the socio-cognitive processes that are at the core of learning and practice, in other words, that “underlie the development of particular perspectives, habits, or skills” (Tay et al., 2018, p. 218). In this study, this was evident primarily in the sense of accomplishment and mastery reported by all participants. Socialization, the third mechanism, is defined as “the degree to which individuals take on various roles and identities within communities and cultures.” The authors highlight how participating in new activities helps build new networks and broaden and develop social roles, which can lead to the formation of new identities and pro-social behavior. The *Lullaby Project* provided opportunities for rich relational dynamics, in a short period of time. From the participants’ accounts, there was evidence of enhanced socialization with peers, with the team and with one’s child (in the case of the mothers group). Participants shared how the experience led to new connections and the strengthening of existing ones. It also fostered new roles both during the project and in prospection. This was evident, for example, in the case of the men’s group, through some participants becoming a radio staff member in the prison and prospective music students, or in the women’s group, through joining other mothers in singing their songs to each other’s children and hoping to one day replicate the same project elsewhere, to pay-it-forward. Crucially, for all participants, the lullaby also functioned as a relational bridge with their families of origin. For example, one father shared how he now has something of value to offer his son when he comes to visit, while some of the mothers emphasized the joy of sharing their lullaby with their family abroad.

Finally, in the context of Tay et al.’s (2018, p. 218) conceptual framework, reflectiveness refers to “an intentional, cognitive-emotional process for developing, reinforcing, or discarding one’s habits, character, values, or worldview.” The authors suggest that engagement with the arts and humanities may trigger insights into aspects of the self, potential desire towards change or even facilitation of that change. This can lead to enhanced critical thinking and perspective-taking. The *Lullaby Project* was lived by participants as a means for a richer perspective about one’s life and one’s children. The process was mentioned for example, as a source of insight into their own sense of resilience and coping, triggering gratitude and strengthening hope and optimism about future plans.

Tay et al. (2018) go further in suggesting that different modes of engagement with the arts may naturally lead to different levels of reflectiveness. We argue that the level of personal investment required for the creation of an individual song, driven by a relational theme (one’s relationship with one’s child), and in the context of adversity, may be particularly fitting for fostering reflectiveness. As the body of research on music and flourishing develops, it will be valuable to further understand how different modes of engagement are experienced. Of interest is also the specificity of the project design and the particular needs of the populations involved in relation to possible mechanisms of flourishing.

### Distinctive Features of the *Lullaby Project*

Several unique features of the project design stand out as possible contributors to the encouraging accounts of change emerging from the data. These are particularly relevant having in mind the specific needs of the communities involved and deserve attention in further projects. First, the *Lullaby Project* has a very short time-span, potentiating quick feedback. Its duration was key for sustaining high motivation, while importantly, being long enough to allow the building of trust and connectedness. Short-term interventions are particularly relevant in these two contexts, considering the typical instability around attendance. It is common for inmates to move to different prisons, in and out of prison, or have conflicting appointments, making it difficult to implement more traditional, continuous programs. Similarly, the refugee group presents typical logistical limitations (e.g., constant changes in housing arrangements, difficulties in affording transport to attend the sessions, need for childcare).

Second, the project places centrality on individuality, while strongly relying on mutuality. Through prioritizing a sense of ownership, it paves the way for the development of stronger agency. Not only is the process highly creative, it fully encourages the expression of the individual’s uniqueness. This appears as a distinctive feature, when comparing with similar projects (e.g., Baker and Mackinlay, 2006) which typically use pre-written songs.

The project is also guided by a strong output, a tangible and high-quality CD recording, very personal and long-lasting. This contributes to continued impact, allowing for the experience to outlive the project duration.

There are also logistical features of the project that contribute to its effectiveness. First, it is relatively portable and easily embedded in each of the partners’ pre-existing structures. Its organic integration was particularly evident in the women’s project, where there was a previously established group of mothers and childcare was provided when needed. Another key aspect was the room for flexibility and adaptation to specific challenges inherent to each particular context (for example, language barriers and the change in the number of participants during the project). Finally, it does not require a large amount of instruments or musical equipment to be executed.

### Limitations and Suggestions for Further Research

Despite the unequivocal positive results of the current study, they stand only as a first step toward the understanding of the potential for impact of the *Lullaby Project’s* model. First, the sample was small, which, despite being desirable for an in-depth study, limits any conclusions on the generalization of findings. The study also did not include a control or a comparative element. These stand as the first suggestions for further research.

Another limitation worth addressing in future studies refers to the potential language barriers in data collection. The current study ensured the possibility of translation when needed. Future studies would ideally consider offering the possibility of being interviewed by a native speaker of the participants’ mother tongue.

Additionally, it will be highly relevant to assess the impact of the project longitudinally, checking for sustainability and transferability of change. Therefore, a follow-up assessment is encouraged. In line with this, the investigation of different project durations and frequency of engagement and their potential influence on flourishing is also of relevance.

The specific contribution of highly individualized composition as a vehicle for creation and expression of meaning also deserves a finer investigation, in particular the process of creating music for one’s child. From the accounts of the participants of this study, there seems to be some distinctiveness around a personal lullaby, which due to its idiosyncrasy possibly represents a stronger output than what traditional community music projects usually offer.

Of particular interest is also the clarification on the specific pathways connecting music creation in this format with the flourishing outcomes, alongside potential individual-level moderators, such as personality, artistic preferences, and skills. The proposal by Tay et al., 2018 stands as a valuable framework for this investigation.

A focus on the children also appears as a natural next step towards a better understanding of the potential of this project, in particular given the known impact of social vulnerability on infants’ language development (Hart and Risley, 2003) and attachment (Miller, 2016) and the role that music can play in potentiating both (Brandt et al., 2012; Vlismas et al., 2013).

Finally, the specific contexts investigated call for further insight on the *Lullaby Project’s* potential as promoter of resilience. The construct stands as both a process and an outcome of positive adaptation to adversity (Masten et al., 1990). It concerns the ability to experience healthy levels of psychological and physical functioning despite highly disruptive events (Bonanno, 2004). Resilience implies both recovery (re-gaining previous levels of functioning that were disrupted) and sustainability over time. Several factors have been evidenced to potentiate resilience, such as positive emotions, self-efficacy, self-esteem, active coping (involving proactivity and planning), optimism, social support, meaning, and purpose in life [see Kent et al. (2013) for a review]. Given the ongoing adversity experienced by both migrants and inmates, along with the potential for this project to elicit key elements for a successful adaptation, it will be valuable to integrate an intentional evaluation of resilience. In particular, it will be highly relevant to explore the link between specific adversities and modes of musical engagement.

In summary, the pilot implementation of the *Lullaby Project* was highly successful in promoting flourishing and represents a relevant model of community-based music interventions for the UK context, in particular for work with migrant and prison populations and for both mothers and fathers alike. Not only was the project experienced as personally significant and a promotor of flourishing for all participants, it also evidenced relevance in relation to the uniqueness of the needs of the groups involved. The results strengthen the case for this model to be widely used within community engagement music teams.

## Data Availability Statement

The datasets generated for this study will not be made publicly available, following the ethical guidelines respected for the project. Requests to access the datasets should be directed to the author.

## Ethics Statement

This study was reviewed and approved by the National Offender Management Service (NOMS) ethics committee. Participants provided their written informed consent to participate in this study.

## Author Contributions

The author conducted all phases of the study and wrote the manuscript.

## Funding

This research was funded by the Arts Council England.

## Conflict of Interest

The author declares that the research was conducted in the absence of any commercial or financial relationships that could be construed as a potential conflict of interest.

## Publisher’s Note

All claims expressed in this article are solely those of the authors and do not necessarily represent those of their affiliated organizations, or those of the publisher, the editors and the reviewers. Any product that may be evaluated in this article, or claim that may be made by its manufacturer, is not guaranteed or endorsed by the publisher.
