# Supplementary material for: Flourishing Through Music Creation: A Qualitative Investigation of the Lullaby Project Among Refugee and Incarcerated Communities
Source: Front Psychol. 2021 Oct 22;12:588905. doi: 10.3389/fpsyg.2021.588905 (PMC8594453; doi:10.3389/fpsyg.2021.588905)
Supplement: Supplementary file 1 [file Table_1.DOCX]

Supplementary Material. Interview schedule

| **Domain** | **Items** |
| --- | --- |
| **Research recap + Ethics** | Recap of the research purpose and ethics debrief |
| **Overall evaluation** | 1. Imagine I did not know anything about this project. How would you describe it to me? 2. What were the highlights of the project? 3. What, if any, were the challenges? 4. Suppose that you were in charge and could make one change that would make the project better. What would you do? |
| **Personal impact** | 1. Tell me about the sessions? How did you usually feel before the sessions? How did you usually feel after the sessions? [probe with specific sessions from diaries and observation data] 2. Tell me about your lullaby [probe with lyrics] 3. How did you feel when you started the project? How do you feel now? |
| **Expectations** | 8. Was the project in line with what you expected? [if applicable: during the sessions, you mentioned [previous project(s)]. How different was this from those other experiences?] |
| **Close** | Ethics review wrap-up (same as #1).  Thank you so much for speaking to me. Before we  finish, do you have any questions or any further  comments you would like to add? |
